# Chloroform in Labor

**Published:** 1893-09

**Authors:** T. Marion Dunagan

**Affiliations:** Barry, Texas


For Texas Medical Journal.

### CHLOROFORM IN LABOR.

BY T. MARION DUNAGAN, M. D., BARRY, TEXAS.

WHEN chloroform was discovered it became to the human family the noblest benefactor the world has ever known; it enables the surgeon to perform the most capital operations with no pain or shock to the patient, while before its discovery they must have been left to die without an operation, or if performed, to succumb to the shock; but now, if the mother, who is the light and joy of a happy home, become the victim of "ovarian tumor" the skillful gynecologist, assisted by chloroform, with almost an omnipotent power can lay his hand on that dreaded disease and say to it, "Thus far shalt thou go and no farther; here and now shall thy devastating progress be stayed," and restore the happy woman to her family and friends and give to her perhaps a long life of sunshine and happiness, and make the very walls of that home ring with cheers and gratitude. This is only one picture expressive of the many thousand that are each day being enacted as the result of the God-given power of chloroform. But still there is another anxious hour of female existence that is far more common than the "ovarian tumor" or any condition that invites the surgeon's skill, a condition that comes to the greatest majority who assume the marriage role which has ever been proverbially cited as the hour of the *greatest* of *mortal* suffering, and it is none other than the period of child-birth; and though some would say 'tis *physiological*, is it not agony? Comparable to no other pain? And the mere fact of its being *physiological* and not pathological, does not in the least mitigate the severity of the pains. While this fact is acknowledged, the great majority of our lying-in patients are allowed to endure the travail of child-birth, the most *excruciating* pain, to the bitter end, while we have that near at hand which would deprive it of its greatest terrors, and when a woman had once borne a child under its influence, she would not possess such a horror for the next, and society would be robbed of its greatest sin—criminal abortion,—and suffering womankind of the many diseases that follow in its wake. We would not ask the victim of disease or accident to submit to an operation the *pain* and *shock* of which would not excel that of child-birth, without the administration of anesthetic.

Now, if by the judicious administration of chloroform we can mitigate or abolish the sufferings of child-birth,—that greatest of *physical anguish*,—and that too with perfect safety, it does appear to me, that in doing so, we confer a blessing on woman the value of which would be hard to estimate. This, I will attempt to show by a resumé of authorities (and not from my limited experience and observation) can be done with entire safety to both mother and child. Why is it that we are so dilatory in using this anesthetic in parturition? Is it because we have a long list of victims that have succumbed during its administration, as does sometimes occur in *surgical* practice? No, it can not be this, for Prof. Playfair has informed us in his work on Obstetrics, 3d Amer. Ed., "That as yet there is no case on record of death during the inhalation of chloroform for *obstetrical*

purposes." No, it is simply because the traditional idea is still maintained that "meddlesome midwifery" is fatal and 'tis wrong and wicked to interfere with the laws of nature. To this specious kind of reasoning we have all listened, and have, perhaps, too often allowed ourselves to remain inactive.

One great objection urged against the use of chloroform in labor is, "that it predisposes to or favors *post*-partum hemorrhage." Prof. Isaac E. Taylor, Professor of Obstetrics in Bellevue Hospital College, New York, remarked at a meeting in the Academy of Medicine, that "in some cases in which it had seemed that the forceps were necessary in order to complete delivery, the moderate influence of chloroform had been sufficient to *stimulate the uterus to action* and render the use of instruments unnecessary. In all cases, therefore, of proposed forceps delivery I first try the *stimulating* effects of chloroform upon the uterus, and if that proves insufficient I then proceed to deliver with the forceps."

So we see from the experience of this experienced author and obstetrician that chloroform not only does not produce *inertia* of the uterus, consequently predisposing to hemorrhage, but instead *stimulates the uterus* to action, and many times prevents the necessity of using forceps, and it is my opinion that chloroform, by preventing nervous shock, allaying nervous irritability and thereby husbanding the strength of the entire system, will prevent rather than cause post-partum hemorrhage. Another objection urged by some is, that "chloroform arrests the pains or *uterine contractions*, and therefore prolongs labor." The authority we have just cited in contradiction of a predisposition to post-partum hemorrhage is *one* in opposition to the objection, for in *stimulating the uterus* you shorten rather than prolong labor.

Dr. H. C. Ghent, of Belton, Texas, in a paper read before the Texas Medical Association on "Anæsthetics in Midwifery Practice," says: "I have never known it to diminish the uterine pains in the least, but on the contrary have witnessed the pains increased in power. That chloroform is a uterine stimulant I do not question, for I have witnessed this happy effect time and again."

Dr. E. T. Rulison, Amsterdam, N. Y., in a paper read before the N. Y. State Medical Association on "The use of chloroform in Labor," says: "It shortens labor and in several ways greatly relieves the attendants."

Dr. Chaigneau in "Journal de Medicine," Paris, says: "Chloroform has no effect upon delivery, or on the production of postpartem hemorrhage, or on any of the conditions of the puerperal states.'' He even maintains that acute or chronic pulmonary affections, diseases of the heart, kidneys, or of the nervous system need not interfere with the cautious administration of this anæsthetic.

And as to the dangers attending its administration in obstetrical practice, Dr. J. F. Baldwin, in Columbus Medical Journal, says: ''Chloroform has been given to hundreds of thousands of lying-in women, and the records may be challenged to authenticate a single fatal result when the drug was administered by a physician.''

Prof. Simpson, in his work on "Anæsthetics,'' says: ''I can state positively I have never seen any serious symptoms which could be traced to the chloroform in any one case, either as affecting the mother or child.''

Ringer says, in his Therapeutics, ninth edition: "Choloroform eases the uterine pains without increasing the danger to mother or child.''

Leeshmum says: ''Given on a handkerchief, in small quantities on the approach of each pain during the second stage, it can never do harm. It thus allays pain and assuages nervous irritability, and in the hands of the skillful practitioner, it is a power for good, and never for evil.''

Henry M. Lyman, Int. Surgery, Vol. 1, as follows: ''Its convenience, its agreeable properties, and the remarkable degree of safety which has attended its exhibition under such circumstances, have all combined to give it the preference before any other anæsthetic.''

Dr. W. R. Reid, (*Glasgow Med. Journal*) in an article on ''Anæsthetics in Midwifery:'' ''The knowledge that the heart or kidney disease exists only makes me more anxious to use chloroform, because in such cases the strain of a severe labor is vastly more dangerous without an anæsthetic than with it.'' He further states, ''that a lying-in woman is peculiarly fitted for escaping the dangers usually connected with anæsthesia; the left ventricle of the heart is considerably hypertrophied, and so less likely to weaken under its action. She is kept in a recumbent position, and so to that extent defended from syncope. The action of the heart is aided by the alternate relaxation and contraction of the uterus, and lastly, the anæsthesia tends to produce anæmia of the brain, whereas the labor pains give rise to an engorgement of that organ. The causes taken together seem to me to account for the absence of fatal results in connection with obstetrical anæsthesia.''

Dr. J. Marion Sims says: ''The efforts of parturition cause cerebral congestion, which counteracts the tendency to syncope from chloroform.''

Dr. Fancourt Barnes, of London, (*British Med. Journal*) says: ''The comparative innocence of chloroform in obstetrical practice is due to the pyhsiological increase of power gained by the heart during pregnancy.

Another beautiful conservatism connected with the use of chloroform in obstetrical practice is the fact that the cerebro-spinal system of the nerves are the first to yield to the influence of an anæsthetic, while the ganglionic or sympathetic system are the last to succumb. The womb, being supplied from the hypogastric ganglia of the sympathetic system, would be the last to receive the effects, consequently the contractile power of the uterus would not be interfered with, and the progress of labor would not be retarded. But the neck of the uterus and vagina are supplied by both systems of nerves, hence would come under its influence sooner than the body and fundus; so when given during the second stage, instead of prolonging labor, it would in this way assist dilatation and hasten its termination. And, furthermore, the perineum being supplied from the spinal system of nerves, would be the first to yield to the influence of an anæsthetic, hence these tissues would relax, and the possibility of perneal tears wonld be lessened.

One of the authorities above cited (Ghent) says in reference to its powers to prevent perineal ruptures: "By the use of chloroform I am confident the gynecologist is prevented from surgical interference.''

Another (Baldwin): ''It reduces the liability to rupture of cervix and perineum.''

Still another (Rulison): ''It reduces the number of perineal tears to minimum."

And innumerable are the distinguished authorities I could record, to say nothing of the many thousands of lesser light and notoriety, to plead for the more frequent use of this anæsthetic in natural labor. And I have not presented an array of my own limited experience, but have striven to select from the superabundance of authorities in support of its use those most positive and direct. Ever since the days of the immortal Simpson obstetricians have been persistently urging its use in obstetrical practice, but the profession, with its inherent conservatism, has been slow to forsake the beaten paths, and it will only be by "line upon line and precept upon precept" that the desired end can be attained.

So let us as physicians living in a scientific era utilize this glorious gift for the amelioration of those "incomparable pains" incident to childbirth, and by so doing, destroy to a great extent that great horror of childbearing felt by all women.

And, as is aptly said by one writer, "bring increased respect for the medical attendant, and the gratefulness depicted upon the countenance of the woman when informed that she is a mother (having become so without pain), cannot fail to arouse in him thoughts so pleasing that he is apt to forget for a moment that a doctor has any trials.

